# Usability and Satisfaction of Augmented Reality During Pediatric Anesthesia Induction: A Pilot Study

**DOI:** 10.1089/jmxr.2023.0009

**Published:** 2024-03-19

**Authors:** Christopher T. Lin, Arianna S. Lane, Erin T. Annick, Margaret J. Klein, Jennifer Lau, Abhishek Karnwal, Maria Fritock, Jeffrey I. Gold

**Affiliations:** 1 Keck School of Medicine, University of Southern California, Los Angeles, California, USA.; 2 Department of Anesthesiology Critical Care Medicine, The Saban Research Institute at Children's Hospital Los Angeles, Los Angeles, California, USA.; 3 Department of Anesthesiology, New York Presbyterian Hospital Weill Cornell Campus, New York, New York, USA.; 4 Department of Psychology, University of Denver, Denver, Colorado, USA.; 5 John A. Burns School of Medicine, University of Hawaii at Manoa, Honolulu, Hawaii, USA.; 6 Department of Anesthesiology, Pediatrics, and Psychiatry and Behavioral Sciences, Keck School of Medicine, University of Southern California, Los Angeles, California, USA.; 7 Department of Anesthesiology, Pediatrics, and Psychiatry and Behavioral Sciences, Keck School of Medicine, University of Southern California, Los Angeles, California, USA.

**Keywords:** pediatric, induction, anesthesia, augmented reality

## Abstract

The induction of anesthesia in children is a frightening experience. Augmented reality (AR), a head-mounted gaming experience, may increase cognitive load and reduce overall distress in children during induction. The study investigates the satisfaction and usability of AR during induction from the perspectives of the patient, caregiver, and health care provider. In this pilot study, 24 patients aged 10–19 years underwent an AR intervention during anesthesia induction. Patients, caregivers, and health care providers completed pre- and postprocedural surveys, including the anxiety thermometer visual analog scale (VAS), the Gold & Rizzo Immersion and Presence (GRIP) inventory. The primary outcome was determined by conducting univariate analyses and evaluating qualitative data to gauge the overall usability and satisfaction of AR. Patients, caregivers, and health care providers were highly satisfied with the AR intervention. Eighty-eight percent of health care providers reported the AR game helped patients during induction. Caregivers rated the AR's effectiveness in reducing patient anxiety at a mean of 9.25 out of 10. Patients rated AR's helpfulness 9.1 out of 10 and calming effects 8.8 out of 10. Patients did not experience an increase in anxiety during induction relative to baseline levels based on VAS ratings (*p* = 0.102). The GRIP inventory demonstrated that AR had moderate to high satisfaction, immersion, and functionality. Patients, caregivers, and health care providers supported the usability and calming benefits of AR with high levels of satisfaction. The intervention is immersive, fun, and easy to use, with the ability to engage patients during pediatric anesthesia induction. The trial was registered before patient enrollment at clinicaltrials.gov (NCT04268914, Principal Investigator: J.I.G.., Date of Registration: October 22, 2019).

## Introduction

More than 3 million children undergo ambulatory surgery in the United States annually.^1^ The induction of anesthesia is potentially the most stressful event that a child experiences during the entire perioperative period.^2^ Younger children experience increased perioperative anxiety.^3^ In addition, anxiety during anesthesia induction is known to cause adverse postoperative behavioral effects, including a higher incidence of emergence delirium.^4^ Sedative premedication, such as midazolam, is used to reduce distress. However, it can delay a patient's recovery and discharge.^5^ Therefore, nonpharmacological methods to improve the patient experience during the induction process have significant preoperative, perioperative, and postoperative implications.

Emerging research in anesthesiology has increasingly focused on nonpharmacological interventions, such as video games on a tablet, cartoons, and more, to enhance the patient experience during induction. Interactive interventions such as video games, when compared with more passive techniques, have demonstrated greater effectiveness in reducing anxiety during induction, due to the cognitive and motor absorption required by the activity.^6^

In this era of technological advancement, a new tool has been introduced to the medical space: augmented reality (AR). AR is a technology that uses a smartphone and special glasses to overlay a computer-generated image onto the real world. For example, a patient in the operating room (OR) can see the medical setting and health care providers around him/her in addition to the AR's animated figures. Most recently, AR has been used within the pediatric space to distract patients during intravenous access, dental care, burn care, and oncological care.

Physicians have also begun to use AR through AR-assisted ultrasound guidance for procedures in which anatomical orientation is important, such as central venous catheter placement, joint injections, and spinal anesthesia.^7^ In the preoperative setting, AR has been used to teach relaxation techniques that have resulted in reductions in anxiety during anesthesia induction.^8^ To date, only a single case report has examined AR as a distraction mechanism to reduce anxiety in children undergoing anesthesia induction, for which there is outstanding potential.^9^

A similar technology, virtual reality (VR), utilizes a headset to create a simulated environment separate from the real world. VR improves medically related pain and anxiety during routine procedures, such as blood draws.^10^ In recent studies, patients who used VR during general anesthesia induction reported less anxiety than those not using VR.^11,12^ Despite having similar names, AR and VR have entirely different functions: VR isolates users in a computer-generated environment, whereas AR enhances the user's perception of their real surroundings with digitally overlaid animations.

Because of this distinction, AR has numerous advantages over VR in the context of anesthesia induction. In contrast to the enclosed VR headset, the open AR chassis permits easier access to the nose and mouth, comfortably fitting a mask for induction or preoxygenation. The clear visor of the AR headset is also beneficial for both the patient and health care provider; the transparency of the visor allows health care providers to observe the patient's facial expressions and easily test the eyelash reflex.

Furthermore, games played in an AR environment can engage patients' attention without impeding awareness of their surroundings in the OR. VR has a strong evidence base, whereas AR is a growing area of research that is promising but needs more evidence.

The purpose of this study was to determine the usability and satisfaction of AR in conjunction with standard of care in pediatric patients undergoing anesthesia induction for ambulatory surgery. The secondary outcome of the study was to gauge the effects of AR on patient anxiety as well as the influence of midazolam on the AR experience. The usability of AR was explored through qualitative and quantitative reports from the patient, caregiver, and health care provider. We hypothesized that the AR intervention would successfully immerse patients during pediatric anesthesia induction and be highly satisfactory, engaging, and easy to use.

## Methods

This study was approved by the Children's Hospital Los Angeles (CHLA) Institutional Review Board (CHLA-15-00461) and written informed consent, assent, and permission were obtained from patients and caregivers participating in the trial. Patient recruitment occurred from October 2019 to March 2020. This article adheres to applicable CONSORT guidelines.

### Procedure

This experimental pilot study includes data from 24 pediatric patients (58% girls; 42% boys; median age = 13 years), their caregivers (parents or legal guardians), and their health care providers (anesthesiologist or certified registered nurse anesthetist who conducted induction). Patients were eligible if they were scheduled for a surgery with general anesthesia in the ambulatory surgery center (ASC) and were between the ages of 10 and 19 years.

Patients were ineligible if they had an American Society of Anesthesiologists (ASA) status of IV or V, had a history of seizures, had a disability or developmental delay, took pain or anxiety medication, had a visual or auditory deficit that would hinder their ability to play the AR, or if, on that day, they had flu-like symptoms. Both English- and Spanish-speaking families were eligible to participate. Regarding ethnicity and medical status, the current sample is consistent with the general CHLA population.

All study participants received the AR intervention. Patients, along with CHLA standard care (CHLA SC) protocol, received the added component of AR technology through the Mira Prism. Current standard of care practices for anxiolysis in outpatient surgery include receiving midazolam, if needed, or other interventions chosen by the health care provider.

The Mira Prism is a portable AR head-mounted display powered by iPhone. When paired with an iPhone, the Mira Prism headset can superimpose computer-generated images on the user's view of the real world. Unlike the full immersion of VR, AR allows users to view the outside world and easily communicate with health care providers while interacting with digital content. In this study, the patient interacted with a game called Magic Mallet (developed by Miney Moe™).

In the game, asteroids in a starry sky fly toward the user, who uses a single hand controller and a flick of the wrist to explode the rocks into a flurry of sparkles. Magic Mallet is a game designed for pain management while preserving communication channels during procedures. It has been previously studied for pediatric otolaryngology procedures as a tool to reduce fear and promote cooperation.^13^ The game automatically reorients itself, so patient movement on the gurney does not interrupt gameplay.

**FIG. 1.**
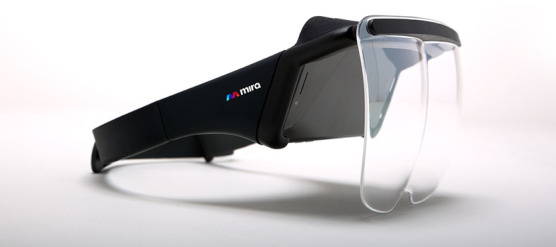
Mira Prism AR headset. The Mira Prism AR headset uses an iPhone to generate a computer-generated image across the transparent visor of the headset. Patients can visualize their surroundings in the OR while also interacting with the overlaid images in the AR game. AR, augmented reality; OR, operating room.

Patients can play the game in an upright or supine position, allowing repositioning with ease. Based on a patient's self-report of anxiety on a 0–10 scale during gameplay within the app, the game adjusts (higher scores of anxiety = increased game acceleration) cognitive load by means of a faster pace and additional asteroids for optimal effect on users. An image of the AR headset can be seen in Figure 1 and a point of view video of the AR intervention can be seen in Supplementary Video S1.

Patients began interacting with the AR environment ∼1–2 min before OR transport. Patients engaged with the game while being transported to the OR and during the anesthesia induction procedure, until falling asleep, at which point, the headset was removed. All patients received preoxygenation through face mask and anesthesia induction intravenously.

### Measures

Patients, caregivers, and health care providers completed pre- and post-questionnaires to assess the AR intervention and their perception of its effectiveness in engaging patients during anesthesia induction (in the form of 10-point scales and yes–no questions).

Patients completed the anxiety thermometer VAS to measure anxiety before and during induction. The VAS anxiety measure is a vertical VAS, anchored with 0 at the bottom indicating the least amount and 10 at the top indicating the greatest amount, measuring a patient's response to “how nervous, afraid, or worried” they were about the upcoming induction. The scale has color cues, graded from yellow at the bottom to dark red at the top, as well as a neutral face at the bottom and a face showing a negative expression at the top. VAS scales demonstrate strong validity and reliability for measuring anxiety in children.^14^

Patients also completed the Gold & Rizzo Immersion and Presence (GRIP) inventory.^15^ The GRIP inventory is a 16-item Likert-like measure used to assess the child's depth of immersion, the believability of the AR intervention, and the functionality of the AR system. Items assess the child's sense of involvement, realism, and transportation into the experience.

### Data collection

Patients completed questionnaires through Qualtrics on iPads at two time points: a preprocedural baseline survey (VAS) in the waiting room after consent and the postprocedural battery (GRIP, additional AR questions, VAS) in the postanesthesia care unit (PACU). Postprocedural questionnaires asked patients to recall their experience leading up to the induction procedure.

Caregiver study participation followed the same timetable as the patient. Of note, caregivers were present for OR transport, but not for the induction process in the OR.

Immediately after induction, the health care provider completed a survey. The health care provider survey is a six-item Likert-like self-report measure of their perceptions about the child's cooperation and anxiety surrounding the induction procedure and overall satisfaction related to the induction procedure. Patients, caregivers, and health care providers were invited to write comments about the use of AR.

### Statistical analysis

All data collected were entered, coded, scored, and analyzed using SPSS. Patient characteristics, demographics, and study variables were summarized using mean (standard deviation [SD]), median (interquartile ranges [Q1, Q3]), or frequency (%), unless otherwise noted. Nonparametric-related samples *t*-tests (Wilcoxon signed rank test) were conducted to compare differences in anxiety at baseline and during the induction procedure.

Nonparametric independent sample *t*-tests (Mann–Whitney *U*) were conducted to compare differences in anxiety and AR immersion scores between patients who did or did not receive preoperative sedatives. Little is known about the SD of anxiety and AR satisfaction, immersion, functionality; therefore, we sought to enroll at least 20 patients to explore these measures.

## Results

**FIG. 2.**
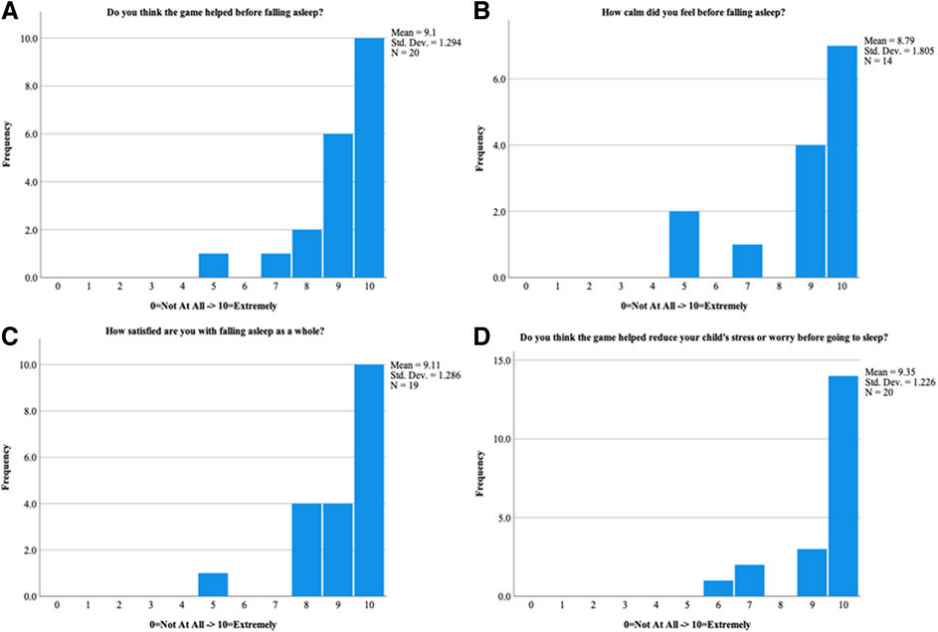
AR anxiety reduction effectiveness ratings. **(A)** AR induction helpfulness rating by child (*μ* = 9.1, *σ* = 1.3, *n* = 20) when patient was asked if game helped with falling asleep. **(B)** AR induction anxiety reduction rating by child (*μ* = 8.8, *σ* = 1.8, *n* = 14) when patient was asked if they felt calm before induction. **(C)** AR induction overall satisfaction by child (*μ* = 9.1, *σ* = 1.3, *n* = 19) when patient was asked about satisfaction with induction process. **(D)** AR induction anxiety reduction rating by caregiver (*μ* = 9.4, *σ* = 1.2, *n* = 20) when caregiver was asked about patient's stress level before induction.

A total of 24 triads (patient, caregiver, and health care provider) were enrolled, and patient participants consisted of 58% girls with an overall median age of 13 years (Table 1). Data demonstrated that patients, caregivers, and health care providers had high rates of satisfaction and engagement with the AR intervention. Patients rated the AR's effectiveness in helping them during induction at an average of 9.10 out of 10 (SD = 1.29), and AR's calming effect before falling asleep at an average of 8.79 out of 10 (SD = 1.81) (Fig. 2A, B).

**Table 1.** Demographics and Baseline Characteristics

*n = *24	*n *(%)
Gender	
Female	14 (58)
Age	
10–12 Years	10 (42)
13–19 Years	14 (58)
Median (Q1, Q3)	13 (11, 16)
Ethnicity	
Hispanic/Latino	14 (58)
White/non-Hispanic	4 (17)
African American	0 (0)
Asian/Pacific Islander	2 (8)
Other (includes multiracial)	4 (17)
Baseline anxiety (VAS) median (Q1, Q3)	3.19 (2.42, 5.79), *n* = 23

VAS, visual analog scale.

Fourteen out of 14 (100%) patients who completed the postprocedure questionnaire answered “yes” when asked if the AR game helped them feel calmer during the induction process. Fourteen out of 19 (73.7%) patients claimed that they did not have any distress before induction. Children were highly satisfied with the overall induction process with a mean of 9.11 out of 10 (SD = 1.29) (Fig. 2C).

The AR intervention also made a positive impression upon caregivers and health care providers. Caregivers rated the AR's effectiveness in reducing their child's anxiety and distress before induction at a mean of 9.35 out of 10 (SD = 1.22), with a minimum reported score of 6 out of 10 (Fig. 2D). Meanwhile, 21 out of 24 health care providers (88%) reported that the AR game helped the child fall asleep, and 22 out of 24 health care providers (92%) would use the AR game again. In answering both questions, none of the health care providers responded negatively; only two (8%) felt “unsure” about the impact of AR on induction.

The GRIP inventory revealed that the AR system had moderate to high levels of satisfaction, immersion, and functionality. The GRIP had composite subscores for satisfaction (5.11 out of 8), immersion (9.86 out of 14), and functionality (8.94 out of 10) (Appendix Table A1).

**FIG. 3.**
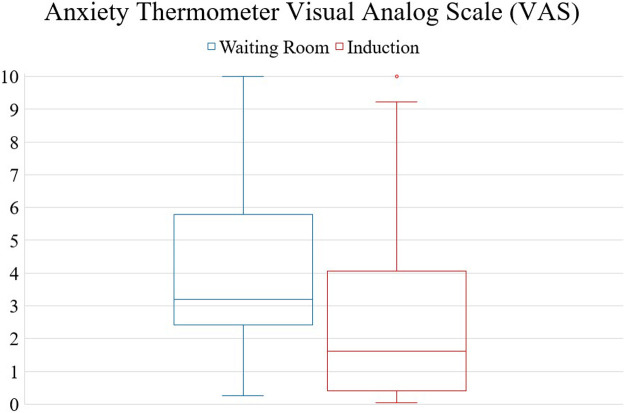
Induction anxiety measure outcomes. Child anxiety thermometer VAS at baseline (median = 3.19, [2.42, 5.79], *n* = 23) versus during induction (median = 1.62 [0.4, 4.05], *n* = 20). VAS anxiety has a maximum score of 10 and induction ratings were obtained after the procedure in the PACU. PACU, postanesthesia care unit; VAS, visual analog scale.

Before induction, patients' median anxiety was a 3.19 out of 10, and during induction, patients' median anxiety rating, which was retrospectively assessed in the PACU, was 1.62 (Fig. 3). There was no significant difference in mean anxiety scores before and during induction of general anesthesia (*p* = 0.102).

In line with standard of care, 12 of 24 patients were given preoperative sedatives before OR transport. There were no significant differences in patients' ratings of overall induction experience and satisfaction, AR helpfulness, GRIP satisfaction, and GRIP functionality for those who did or did not receive preoperative midazolam (Appendix Table A2). There was a significant difference between GRIP Immersion scores (*p* = 0.04). Moreover, there was no significant difference in patient cooperativity as rated by health care providers among patients who did or did not receive midazolam (*p* = 0.71).

No participants experienced or reported adverse events due to the AR intervention, including dizziness or nausea. In one case, the AR headset needed minor adjustments, whereas the mask for preoxygenation was placed in the OR, which momentarily delayed the start of the procedure.

## Discussion

The hypothesis that AR in conjunction with standard of care would be usable and well-liked in pediatric patients undergoing induction of anesthesia is supported by current findings. There is a strong perceived benefit of AR as a fun and engaging mechanism as reported by patients, caregivers, and health care providers alike. In addition, there is no significant increase in anxiety during anesthesia induction compared with preprocedural baseline, indicating that the AR intervention in conjunction with SC may prevent increases in anxiety during induction.

Our findings display the benefits of a tailored AR game, Magic Mallet, for the medical space. Patients find the game to be moderately to highly enjoyable, immersive, and easy to use. These positive attributes support the potential of an AR game to engage the cognitive load of children as they undergo anesthesia with fun visuals and audio. Specifically, Magic Mallet adjusted for the anxiety level of the patient, providing an individually customized induction experience. Most notably, the game has a high functionality score, indicating that patients can easily learn, play, and benefit from the game.

Qualitative evidence also exhibits the unique benefits of AR for anesthesia induction. One health care provider states, “It is definitely beneficial to most patients who are first timers to surgery/anesthesia to create distractions especially during IV insertions or important parts of the care like the induction phase.” An 18-year-old patient describes his thoughts as he was falling asleep: “I was nervous, because I didn't see the surgery room before. But, also calm, because I was playing the game.” With its high ratings in satisfaction and calmness, AR has the potential to change the preoperative and perioperative climate such that a child goes from distressed to comfortable.

Based on the questionnaire results, anesthesiologists were satisfied with the ability of AR to help children fall asleep during induction and to keep them calm. The improved OR environment and throughput can easily extend the satisfaction to other health care staff in the preoperative, OR, and postanesthesia space. Owing to its small form factor and straightforward mechanics, this study's version of an AR headset can be integrated into the perioperative space without complications.

It is as simple as remembering to put on the AR headset before transporting the patient to the OR and removing the headset after anesthesia induction is complete and the patient is unconscious. With current concerns surrounding pharmacological usage in pediatric patients, AR offers an interactive and fun immersion mechanism that can be easily implemented in the OR and further studied.

Of note, our study included two patients with attention-deficit/hyperactivity disorder (ADHD) and one patient with ADHD and autism. Although 2 of the 3 patients received midazolam, the mean helpfulness rating among the 3 patients is 7 out of 10, which is lower than the sample population mean of 9.1. One of the patients, a 10-year-old, states “It was pretty cool… I think it would help other kids but it wasn't very helpful for me because I was thinking about everything else.” The sample size of patients with ADHD and autism in this study is limited.

Nonetheless, this patient's statement exemplifies the varied nature of individual responses to AR immersion and engagement and its potential exacerbation of anxiety in different patient populations. Ultimately, further study is necessary to determine whether AR is helpful for neurodivergent patients.

Using a preoperative sedative had minimal effect on the efficacy of the AR intervention. Besides a minimal increase in immersion in the AR game, patients who did and did not receive midazolam had similar ratings on the overall induction experience, helpfulness of AR, GRIP satisfaction and functionality scores, cooperation, and anxiety scores.

These data demonstrate that when AR is used, midazolam may not improve the overall induction experience, reduce anxiety, or increase cooperation during anesthesia induction. However, a more controlled study is needed to further explore the role of midazolam during AR gameplay before induction, as many factors play into a health care provider's decision to administer preoperative sedatives to a patient.

This study details the first known interactive AR game for pediatric patients undergoing induction. A recent case report illustrated the usability of AR for pediatric anesthesia induction in which a cartoon robot encouraged patients to remain calm and take deep breaths.^9^ Our study improves upon the immersive nature of the case report through the integration of a single-handed controller and the cognitive adjustment of the game's interactions based on anxiety scores.

Also, a randomized clinical trial has demonstrated the efficacy of a VR game for anxiety reduction during pediatric anesthesia induction;^11^ though, the superior functionality benefits of the AR headset, with which there is a more open interface between the patient, health care provider, and the OR surroundings, have been described earlier. The current investigation improves and expands on the previous studies with an AR game that engages and immerses the patient while maintaining their awareness of the OR. Emerging research in this space indicates strong potential for innovative and immersive nonpharmacological interventions to improve the induction experience.

This study has some limitations. First, patients completed retrospective measures on their induction experience in the PACU, after their completed surgery. Retrospective time frame could occur anywhere from 30 min to several hours after the procedure. Patients may have recall bias or amnesia as a direct result of the anesthesia. Moreover, waking up and realizing that the surgery was over and successful could negatively skew anxiety scores, falsely suggesting lower anxiety.

In addition, patients could have rated AR positively because investigators were present during the survey completion period in the PACU, resulting in potential observation bias. Next, although previous studies unrelated to anesthesia induction have used AR in children as young as 8 years old, the large AR headset used in this study made it difficult to implement on children younger than 10, a critical population of great concern in the perioperative space.^8^

These younger pediatric patients may have vastly different responses to the AR intervention, in the same way that younger patients often experience more perioperative anxiety when compared with the current population in this study.^3^ The demographic distribution of the study population seen in Table 1 is representative of the patient population at CHLA.

With this in mind, the small sample size included in the study lacks exploration into broader demographic groups, whether by age or race, and the conclusions drawn in this study may not be generalizable to all pediatric patient populations. Lastly, this is a pilot study and does not include a control sample to compare the effects of AR versus no AR. Therefore, results need to be interpreted carefully.

Future investigations examining the effects of AR for decreasing anxiety associated with induction would benefit from a randomized-controlled design. These studies could examine the anxiolytic effect of AR when compared with no AR, and VR, in the presence or absence of preoperative midazolam. There is also significant potential in comparing AR with other commonly used nonpharmacological interventions such as satisfaction and anxiety reduction versus parental presence or the use of an iPad or iPhone for video distraction.

In addition, with the development of smaller AR headsets, the opportunity to study children below the age of 10 could be vital to anxiety management for anesthesia induction. A study into younger pediatric patient populations is especially important, due to the different methods of anesthesia induction such as intravenous versus mask induction.

Future research can also examine postoperative behavioral implications, including emergence delirium, as an outcome. They can also potentially investigate for differences in outcomes through physiological (heart rate sensors) and biological (cortisol) markers. In pediatric patients with chronic conditions who may undergo procedures frequently, it may be beneficial to look at long-term outcomes regarding the effect of AR on future hospital visits or procedures.

Overall, AR is a promising technology that can be used as a complement to standard of care procedures to engage the attention of children during induction. When paired with SC, AR has notable potential to increase patient, caregiver, and health care provider satisfaction and assistance in patient sleep onset. AR has the unique ability as a nonpharmacological treatment to potentially reduce anxiety, distress, and improve future health outcomes in a fun, interactive, and entertaining manner. Continuing to study the effects of AR in the perioperative space can have a significant ripple effect on every child's OR experience, as well as that of caregivers and health care providers.

## Abbreviations Used

ADHD: attention-deficit/hyperactivity disorder
AR: augmented reality
ASC: Ambulatory Surgery Center
CHLA: Children's Hospital Los Angeles
GRIP: Gold & Rizzo Immersion and Presence
OR: operating room
PACU: postanesthesia care unit
SC: standard care
SD: standard deviation
VAS: visual analog scale
VR: virtual reality

## Appendix

**Appendix Table A1.** Gold & Rizzo Immersion and Presence Inventory for Augmented Reality

Question (0 = no, 1 = a little, 2 = a lot)	0	1	2	No. of responses
Satisfaction				
Did you enjoy the game?	0	4	14	18
Were you sad or disappointed when the game was over?	10	6	1	17
Was the game interesting compared with other computer games you have played?	2	7	9	18
Would you like to play the game again?	1	7	9	17
Immersion				
Did the game grab your attention?	1	4	12	17
Did it feel like you were really there?	4	6	8	18
Were you interested and involved in the game?	0	7	11	18
Did the things you saw look real?	1	11	6	18
Did the way things moved look real?	4	6	8	18
Did the things you heard sound real?	3	6	8	17
Did it feel like you were in control of what happened in the game?	0	7	10	17
Functionality				
Did you find the game easy to play?	0	5	13	18
Were you worried about putting on the headset?^a^	17	0	1	18
Was the headset comfortable when you had it on?	0	7	11	18
Were the controls easy to use?	0	2	16	18
Did you get used to playing the game quickly?	0	4	14	18

	Subscore	Max		
**Satisfaction subscore**	5.11	8		
**Immersion subscore**	9.86	14		
**Functionality subscore**	8.94	10		

a Item was reverse scored when summarizing composite scores.

**Appendix Table A2.** Mann–Whitney *U* Tests Analyzing Midazolam's Influence on Augmented Reality-Associated Induction Experience

Category	Questionnaire	*Significance (*p*-value)*
Overall induction	Patient	0.905
AR helpfulness	Patient	0.766
Induction satisfaction	Patient	0.720
GRIP satisfaction	Patient	0.408
GRIP immersion	Patient	0.040
GRIP functionality	Patient	0.146
Patient cooperation	Health care provider	0.713

AR, augmented reality; GRIP, Gold & Rizzo Immersion and Presence.

## References

[1] Hall MJ, Schwartzman A, Zhang J, et al. Ambulatory surgery data from hospitals and ambulatory surgery centers: United States, 2010. Natl Health Stat Report, 2017;(102):1–15. 28256998

[2] Kain ZN, Mayes LC, O'Connor TZ, et al. Preoperative anxiety in children: Predictors and outcomes. Arch Pediatr Adolesc Med, 1996; 150(12):1238–1245; doi: 10.1001/archpedi.1996.02170370016002 8953995 10.1001/archpedi.1996.02170370016002

[3] Johnson AH, Conley B, Koruthu S, et al. Pediatric preanesthesia anxiety and factors of family satisfaction. J Perianesth Nurs, 2023; 38(2):312–317; doi: 10.1016/j.jopan.2022.06.006 36528451 10.1016/j.jopan.2022.06.006

[4] Kain ZN, Mayes LC, Caldwell-Andrews AA, et al. Preoperative anxiety, postoperative pain, and behavioral recovery in young children undergoing surgery. Pediatrics (Evanston), 2006; 118(2):651–658; doi: 10.1542/peds.2005-2920 10.1542/peds.2005-2920 16882820

[5] McCann ME, Kain ZN. The management of preoperative anxiety in children: An update. Anesth Analg, 2001; 93(1):98–105; doi: 10.1097/00000539-200107000-00022 11429348 10.1097/00000539-200107000-00022

[6] Uyar BS, Polat R, Bolat M, et al. Which is good for pre-operative anxiety? midazolam, video games or teaching with cartoons: A randomised trial. Eur J Anaesthesiol, 2020; 38(7):744–750; doi: 10.1097/EJA.0000000000001384 10.1097/EJA.0000000000001384 33186304

[7] Privorotskiy A, Garcia VA, Babbitt LE, et al. Augmented reality in anesthesia, pain medicine and critical care: A narrative review. J Clin Monit Comput, 2022; 36(1):33–39; doi: 10.1007/s10877-021-00705-0 33864581 10.1007/s10877-021-00705-0

[8] Chamberland C, Bransi M, Boivin A, et al. The effect of augmented reality on preoperative anxiety in children and adolescents: A randomized controlled trial. Pediatr Anesth, 2024; 34(2):153–159; doi: 10.1111/pan.14793 10.1111/pan.14793 37925608

[9] Libaw JS, Sinskey JL. Use of augmented reality during inhaled induction of general anesthesia in 3 pediatric patients: A case report. A&A Pract, 2020; 14(7):e01219; doi: 10.1213/XAA.0000000000001219 10.1213/XAA.0000000000001219 32539270

[10] Gold JI, Mahrer NE. Is virtual reality ready for prime time in the medical space? A randomized control trial of pediatric virtual reality for acute procedural pain management. J Pediatr Psychol, 2018; 43(3):266–275; doi: 10.1093/jpepsy/jsx129 29053848 10.1093/jpepsy/jsx129

[11] Jung MJ, Libaw JS, Ma K, et al. Pediatric distraction on induction of anesthesia with virtual reality and perioperative anxiolysis: A randomized controlled trial. Anesth Analg, 2020; 132(3):798–806; doi: 10.1213/ANE.0000000000005004 10.1213/ANE.0000000000005004 PMC9387568 32618627

[12] Simonetti V, Tomietto M, Comparcini D, et al. Effectiveness of virtual reality in the management of paediatric anxiety during the peri-operative period: A systematic review and meta-analysis. Int J Nurs Stud, 2022;125:104115; doi: 10.1016/j.ijnurstu.2021.104115 10.1016/j.ijnurstu.2021.104115 34781118

[13] Caruso TJ, Madill M, Sidell D, et al. Using augmented reality to reduce fear and promote cooperation during pediatric otolaryngologic procedures. Laryngoscope, 2021; 131(4):E1342–E1344; doi: 10.1002/lary.29098 32886794 10.1002/lary.29098

[14] Bringuier S, Dadure C, Raux O, et al. The perioperative validity of the visual analog anxiety scale in children: A discriminant and useful instrument in routine clinical practice to optimize postoperative pain management. Anesth Analg, 2009; 109(3):737–744; doi: 10.1213/ane.0b013e3181af00e4 19690240 10.1213/ane.0b013e3181af00e4

[15] Gold JI, SooHoo M, Laikin AM, et al. Effect of an immersive virtual reality intervention on pain and anxiety associated with peripheral intravenous catheter placement in the pediatric setting: A randomized clinical trial. JAMA Netw Open, 2021; 4(8):e2122569; doi: 10.1001/jamanetworkopen.2021.22569 34432011 10.1001/jamanetworkopen.2021.22569 PMC8387848

